# Annotations

**Published:** 1905-12-09

**Authors:** 


### ANNOTATIONS.

### Viewing the Corpse.

A DENBIGHSHIRE jury last week flatly refused to comply with the request of the coroner, before whom the inquest was held at Holt, to view the body of a child who had died from diphtheria, the medical man having been called in too late. The coroner was, of course, merely acting in accordance with the provision of an antiquated Act of Parliament, which requires that at an inquest the corpse should be viewed. That at the time the Act was passed there were good reasons for this provision is doubtless true; and although the danger of infection from diphtheria is remote, there ought from the first to have been a clause of exemption in respect to the victims of infectious diseases. The jury at Holt unanimously declined to observe the archaic custom on the ground that they were not going to run the risk of infection. Ultimately it was arranged that they should stand in the garden and look at the body through the window. Obviously, the law was thus reduced to a farce. The remedy, as we said on a former occasion, is that the law should be remodelled, so that a scientific and expert inquiry would replace the futile and cumbersome ritual which is now in vogue. If, however, the absurd system of juries at coroners' inquests is to be maintained, the coroner should have authority to decide as to the necessity for the fulfilment of a function which, in any event, must be painful, is often repulsive, may be fraught with peril, and cannot be of the slightest practical use. We have only to imagine a number of conscientious objectors to vaccination compelled to view the corpse of an individual who died from virulent small-pox in order to realise the folly of continuing a practice opposed alike to modern science and to common sense.

### Figs and Enteric Fever.

A LITTLE while ago some prominence was given in the daily press to an accusation against Smyrna figs. It was stated that in the process of packing the figs sea-water, taken direct from one of the worst parts of the harbour, was used. As the harbour water is subject to the grossest pollution, it was concluded that dried figs packed at Smyrna should be regarded with very active suspicion. On this point, however, the public may now be reassured; for Dr. Reginald Dudfield, medical officer of health for Paddington, at the request of the Public Health Committee of that borough, has had the matter tested by bacterial examination. Sample boxes of figs were purchased at different shops, and forwarded unopened to the Lister Institute of Preventive Medicine. The report on these samples which has now been received, is rather astonishing, for instead of finding an excess of micro-organisms in the figs, there proved to be remarkably few. No bacillus coli or any similar organisms were found in as much as 10 grammes of figs. There is, therefore, no evidence of the least contamination with sewage. When compared with the enormous number of bacteria present in many foodstuffs, and with the not infrequent abundance of intestinal organisms in similar material, these results are somewhat remarkable. The explanation given is that the liquids of these dried figs are in reality strong solutions of sugar. Such solutions are distinctly antiseptic, and therefore the figs undergo a process of sterilisation during and subsequently to the process of drying. Any contamination with organisms akin to the bacillus typhosus is therefore rendered negligible in the natural course of events. Taking it for granted that the examinations were sufficiently numerous to warrant a reliable conclusion, this outcome of the inquiry is highly satisfactory, and it will no doubt be welcome news to the many who consider figs are as inseparable from Christmas fare as roast beef and plum-pudding.

### Compulsory Isolation of Consumption.

SIR WILLIAM BROADBENT'S address at Denison House, Westminster, at the meeting of the Invalid Children's Aid Association, must have given cause for thought to many as to the prevalence of consumption in our midst. He told his audience that since 1901 no fewer than 6,391 children under five years of age had died in London of tuberculosis. South and East London show the worst record in this infantile death-rate, and Hampstead is the most exempt from the scourge, while Kensington and Paddington, in Sir William's opinion, were much worse than they ought to be, largely owing to bad housing conditions. He also dwelt upon the ignorance of the poor as to the proper feeding of children, but we think he did not lay sufficient stress on their ignorance as to the infectious nature of phthisis. That husbands and wives are often infected by each other is well known, and as in the homes of the poor the youngest children often sleep with their parents, they are liable to the most direct form of infection when the father or mother is consumptive. Anything that will bring home to the minds of ignorant people the notion that consumption is an infectious disease, even as scarlet fever is, though it is less immediately fatal, will be a good thing for them and the whole community. Therefore we welcome a statement made by the Local Government Board to a Board of Guardians that inquired on the subject, that advanced tuberculous phthisis, accompanied by expectoration, may be regarded as an infectious disease, and would justify compulsory detention. The Guardians in question seem to have hesitated before accepting the dictum, fearing that the knowledge that he was liable to be detained or removed would prevent a consumptive patient from seeking treatment as soon as he otherwise might. There may be some foundation for this fear, but as a matter of fact very few patients first seek medical aid under the belief that they are suffering from consumption; the ailment passes as a chill, a cough, or something equally vague, and the doctor is generally the first to find out what the trouble really is. As things are, the patient often appreciates his condition only in the later stages, when he has already unconsciously done incalculable harm to those around him. It should be possible to remove him to a proper sanatorium as soon as his condition was discovered, and any apparent hardship involved in this would be atoned for by the possibility that a stay there might restore him to health, and would in any case protect those with whom he lived.

